# Peptide DFCPPGFNTK Mitigates Dry Eye Pathophysiology by Suppressing Oxidative Stress, Apoptosis, Inflammation, and Autophagy: Evidence from In Vitro and In Vivo Models

**DOI:** 10.3390/cimb47060441

**Published:** 2025-06-10

**Authors:** Kaishu Deng, Wenan Li, Jinyuan Liang, Zhengdao Chen, Yan Xu, Jingxi Zhang, Yingtong Zhan, Zhiyou Yang, Shaohong Chen, Yun-Tao Zhao, Chuanyin Hu

**Affiliations:** 1 College of Food Science and Technology, Modern Biochemistry Experimental Center, Guangdong Province Engineering Laboratory for Marine Biological Products, Guangdong Provincial Key Laboratory of Aquatic Product Processing and Safety, Zhanjiang Municipal Key Laboratory of Marine Drugs and Nutrition for Brain Health, Guangdong Ocean University, Zhanjiang 524088, China; dks377736843@163.com (K.D.); wanan000911@163.com (W.L.); jinyy0522@163.com (J.L.); 13399181047@163.com (Z.C.); 18670244665@163.com (Y.X.); 13531083526@163.com (J.Z.); 18218017154@163.com (Y.Z.); zyyang@gdou.edu.cn (Z.Y.); csh3788@163.com (S.C.); 2 Department of Biology, Guangdong Medical University, Zhanjiang 524023, China

**Keywords:** dry eye disease, DFCPPGFNTK, oxidative, apoptosis, inflammation, autophagy

## Abstract

Dry eye is an ophthalmic disease with an intricate pathomechanism, and there are no effective interventions or medications available. We investigated the effects of a peptide, DFCPPGFNTK (DFC), screened from tilapia skin hydrolysate on dry eye and its underlying mechanisms. In vitro, human corneal epithelial cells (HCECs) were challenged by 100 mM NaCl in a hyperosmotic environment. DFC restored the cell viability of HCECs induced by NaCl, reduced the transition of mitochondrial membrane potential, delayed the apoptosis of damaged cells, reduced the production of reactive oxygen (ROS) and malondialdehyde (MDA), increased the activities of superoxide dismutase (SOD) and catalase (CAT), and increased the expression rate of Bcl-2/Bax. Compared to the model group, the protein expression levels of COX-2 and iNOS were down-regulated, the mRNA expression of *Tnf-α* and *Il-6* were decreased, the protein expression levels of Nrf2 and HO-1 were increased, and the levels of autophagy-related proteins p62 and LC3B were regulated. In vivo, the dry eye model was developed by administering eye drops of 0.2% BAC to mice for 14 days. DFC increased tear secretion, changed the morphology of tear fern crystals, prevented corneal epithelial thinning, reduced the loss of conjunctival goblet cells (GCs), and inhibited the apoptosis of mice corneal epithelial cells. In summary, DFC improved dry eye by inhibiting oxidative stress, apoptosis, inflammation, and autophagy.

## 1. Introduction

Dry eye is a common ophthalmic disease that affects 5–50% of the world’s population [1]. There are various causes of dry eye, which usually leads to symptoms such as blurred vision, redness and itchiness, and, even worse, it has a certain impact on the psychological health of the patients, which seriously jeopardizes human life [2]. The pathogenesis of dry eye has still not been fully elucidated, but the vicious cycle is a major feature of the pathogenesis of dry eye, which is inextricably linked to the dynamic imbalance of the tear film, the hypertonic environment of tears, inflammation, and apoptosis [3].

Currently, there are medications that work in various ways in the clinical treatment of dry eye, such as artificial tears, cyclosporine, corticosteroids, etc. But long-term use of these medications has certain shortcomings, which can lead to other uncomfortable side effects in patients [4]. The use of artificial tears provides only partial relief from the symptoms of dry eye. Cyclosporine A may cause stinging and burning.

Natural products have demonstrated significant potential in the management of dry eye syndrome [5]. Polyphenols possess the ability to mitigate oxidative stress and inflammation. For instance, resveratrol has been shown to alleviate dry eye symptoms by reducing both inflammation and oxidative damage to the ocular surface [6,7]. Quercetin, a flavonoid compound, exhibits notable anti-inflammatory and antioxidant properties, garnering considerable attention in the treatment of ocular surface disorders [8]. It has been demonstrated that curcumin exerts a beneficial effect on dry eye syndrome through its anti-inflammatory properties [9].

Bioactive peptides have a broad application prospect in the field of medicine due to their high specificity for target tissues, low accumulation in organisms, and easy degradation in the environment [10]. In recent years, peptides have potentially contributed to the improvement of ocular dysfunction. Cell Penetrating Peptides (CPPs) are considered to have potential in ophthalmic delivery due to their specific structural domains [11]. Pigment epithelium-derived factor (PEDF) has been reported to have an ameliorative effect on dry eye [12,13]. Our group has previously demonstrated tilapia skin peptide (TSP) has anti-dry-eye activity, a mixture of peptides enzymatically prepared from tilapia skin [14,15]. The peptide DFCPPGFNTK (DFC), characterized by the specific sequence Asp-Phe-Cys-Pro-Pro-Gly-Phe-Asn-Thr-Lys, was identified from TSP in silico methods. Following virtual screening analysis, DFC exhibited low toxicity and hypoallergenicity relative to other peptides. As a peptide derived from natural products, DFC exhibits excellent biocompatibility, making it a promising candidate for the treatment of ocular surface disorders, including dry eye. Nevertheless, the specific activity validation and molecular mechanisms of the DFC remain to be fully elucidated.

To further explore the anti-dry-eye mechanism of specific peptides in tilapia skin hydrolysate, DFC was screened and its role in ameliorating dry eye further analyzed in vivo and in vitro from multiple perspectives.

## 2. Materials and Methods

### 2.1. Reagents

Modified Eagle’s medium (MEM, 11095080), fetal bovine serum (FBS, 1907422), and penicillin–streptomycin solution (2289325) were obtained from Gibco (Grand Island, NY, USA). Methylthiazolyldip-henyl-tetrazolium bromide (MTT) was purchased from Shanghai Yuanye Biotechnology Co., Ltd. (Shanghai, China). 2,7-dichlorodihydrofuorescein diacetate (DCFH-DA, C2938) was taken from molecular probes (Carlsbad, CA, USA). SOD, MDA, and CAT kit were purchased from Jiancheng Institute (Nanjing, China). Benzalkonium chloride (BAC, 63449-41-2) was bought from Sigma (St. Louis, MO, USA). Sodium Hyaluronate (SH, H20150150) eye drops were obtained from URSAPHARM Arzneimittel GmbH (Saarbrücken, Germany). Tear test phenol red cotton thread was obtained from Tianjin Jingming New Technological Development Co., Ltd. (Tianjin, China). The terminal deoxynucleotidyl transferase dUTP nick end labeling kit (TUNEL, E-CK-A321) was obtained from Elabscience Biotechnology Co., Ltd. (Wuhan, China). Annexin V-FITC Apoptosis Assay Kit (C1062M), JC-1, Periodic Acid-Schiff Staining Kit (PAS, C0142S), hematoxylin and eosin staining Kit (H&E, C0105S), and BCA Protein Quantification Kit (P0012) were obtained from Beyotime Biotechnology Co., Ltd. (Shanghai, China).

Anti-cyclooxygenase-2 (COX-2, 12282, 1:1000), the antinuclear factor E2-related factor (Nrf2, 20733, 1:1000), SQSTM1/p62 (5114, 1:1000), and β-Actin (4970, 1:1000) antibodies were obtained from Cell Signal Technology (Danvers, MA, USA). Anti-inducible nitric oxide synthase (iNOS, SC-7271, 1:1000) antibody, anti-B-cell lymphoma-2 (Bcl-2, SC-7382, 1:700), and anti-Bcl-2-associated X (Bax, SC-7480, 1:700) antibodies were obtained from Santa Cruz (Dallas, TX, USA). The antiheme oxygenase-1 (HO-1, ab68477, 1:1000) antibody was bought from Abcam (Cambridge, UK). The LC3B (NB100-2220, 1:1000) antibody was bought from Novus Biologicals (Centennial, CO, USA). Immobilon™ Western Chemiluminescent HRP Substrate (WBKLS0100) and polyvinylidene fluoride (PVDF) membranes (IPVH00010) were obtained from Millipore (Bedford, MA, USA).

### 2.2. Synthesis of Cytoprotective Peptides

The discovered peptides were synthetically produced utilizing the Fmoc solid-phase process using 2-CL resin as the amino-acid-binding carrier, given by TGpeptide Biotechnology Co., Ltd. (Nanjing, China). The purity of DFC was determined to be 98.48% via HPLC analysis.

### 2.3. Animals

C57BL/6 male mice (6–7 weeks old) were obtained from Guangdong Medical Laboratory Animal Center (Guangzhou, China). Animal experiments were conducted in strict accordance with the requirements of the Animal Ethics Committee of Guangdong Ocean University (approval number: 2022062101) and the Guidelines for the Management and Use of Laboratory Animals of Guangdong Ocean University. Animals were acclimated for a minimum of one week prior to the study and maintained under standard conditions, including a 12 h light/12 h dark cycle, appropriate humidity, and controlled temperature in a specific pathogen-free environment, with ad libitum access to food and water. Following acclimation, mice were randomly allocated to experimental groups.

### 2.4. BAC-Induced Dry Eye Model and Treatment

Mice were randomly assigned to five groups: control, model, DFC treatment 1, DFC treatment 2, and positive drug control, with 10 mice per group. A mice model of dry eye was established by administering 0.2% BAC twice daily for 14 days [16]. During the treatment phase, mice in the control and model groups received 5 µL of saline per eye; mice in the DFC treatment 1 group received 5 µL of 0.01% DFC per eye; mice in the DFC treatment 2 group received 5 µL of 0.05% DFC per eye; and mice in the positive drug control group received 5 µL of a commercially available 0.1% SH eye drop solution (preservative-free) per eye three times daily for 14 days.

### 2.5. Tear Production and Tear Ferning Test

The procedure began with the anesthetization of the mice. Subsequently, a phenol-red-impregnated cotton thread was placed into the palpebral conjunctiva of the lower eyelid for 30 s. The length of the cotton thread stained red was recorded and served as a quantitative measure of tear secretion in the mice. For the tear ferning test, tear samples from both eyes were collected and evenly spread onto glass slides. These samples were left to air dry at room temperature for three hours. The resulting fern-like crystallization patterns were examined microscopically and evaluated using a previously established grading system [17].

### 2.6. H&E Staining of the Corneal Epithelium

The ocular specimens were fixed in 4% paraformaldehyde (PFA) dissolved in PBS for 24 h. Following fixation, the tissues were embedded in paraffin and sectioned into 5 μm thick slices. Hematoxylin and eosin (H&E) staining was initially performed on these sections to evaluate histological morphology. Corneal epithelial thickness was quantitatively assessed based on H&E-stained images. For each eye, three representative sections were analyzed, and central corneal thickness measurements were obtained using ImageJ software version 1.8.0 (National Institutes of Health, Bethesda, MD, USA).

### 2.7. PAS Staining of Goblet Cells in the Conjunctiva

Paraffin sections were stained in accordance with the instructions provided with the PAS kit and were utilized to ascertain the number of goblet cells in the conjunctiva. The number of GCs in the whole conjunctiva from each eye in three sections was counted using Image pro plus 6.0 software (Media Cybernetics, Silver Spring, MD, USA).

### 2.8. TUNEL Assay

The TUNEL assay was employed to assess apoptotic cell counts in the corneal epithelium of mice, following the manufacturer’s protocol. In brief, paraffin-embedded tissue sections were subjected to dewaxing and rehydration. Proteinase K (100 µL) was applied to each section and incubated at 37 °C for 20 min. After washing with PBS, the sections were treated with 100 µL of terminal deoxynucleotidyl transferase (TDT) equilibration buffer and incubated for 20 min at 37 °C. Subsequently, the TDT equilibration buffer was aspirated, and 50 µL of labeling working solution (prepared in a ratio of TDT equilibration buffer/label solution/TDT enzyme = 7:2:1, *v*/*v*/*v*) was added, followed by incubation in a dark chamber at 37 °C for 60 min. The sections were then rinsed three times with PBS. Apoptotic cells in the corneal epithelium were visualized under a fluorescence microscope (Leica, Wetzlar, Germany) after nuclear staining with DAPI (4,6-diamidino-2-phenylindole). Representative fluorescence images of the central corneal epithelium were captured for each section.

### 2.9. Cell Culture and Treatment

HCECs were purchased from Guangzhou Jennio Biotech Co., Ltd. (Guangzhou, China). HCECs are normal human corneal epithelial cells isolated from dissected limbal sections, the progenitor-rich region where the sclera and cornea join, and were subsequently immortalized using SV40 T-transformed by the provider. The HCECs were cultured in MEM medium supplemented with 10% fetal bovine serum and 1% penicillin–streptomycin solution in a humidified atmosphere of 5% CO_2_ at 37 °C. HCECs were inoculated into 96-well or 6-well plates and incubated for 24 h. The dry eye model in vitro was formed with reference to our previous study; this involved the creation of a hypertonic environment with the addition of 100 mM NaCl [15]. The cells were subsequently treated with DFC for 12 h, with or without 100 mM NaCl.

### 2.10. Cell Viability Assay

After treatment, the MTT assay was employed to assess the cell viability of HCECs. The media was eliminated, and the cells were rinsed thrice with phosphate buffer solution (PBS, pH 7.2). A total of 100 µL of MTT solution (0.5 mg/mL, prepared in PBS) was applied to each well and incubated at 37 °C for 4 h. The supernatant from each well was discarded, and 150 µL of dimethyl sulfoxide was introduced to each well to solubilize the formazan. The data were acquired by measuring the absorbance at 490 nm using a microplate reader (BioTek, Winooski, VT, USA).

### 2.11. Intracellular Reactive Oxygen (ROS) Analysis

ROS were determined according to the reagent company’s instructions. HCECs were plated in 6-well culture plates and subjected to treatments with DFC and NaCl. After a 12 h incubation, the medium was carefully aspirated, and 10 μM DCFH-DA was introduced to each well. The plates were incubated at 37 °C for 30 min, followed by three washes with PBS to remove excess dye. Fluorescent images were acquired using a fluorescence microscope (Leica, Wetzlar, Germany), and ROS mean fluorescence intensity was quantified using ImageJ software.

### 2.12. Flow Cytometry Analysis of Apoptosis

After 12 h of exposure, cultures were collected and HCECs grown in 6-well plates were harvested according to the kit instructions and centrifuged to discard the supernatant, followed by centrifugation with a pre-cooled PBS wash. The cell pellet was resuspended in 1 mL of 1× binding buffer and centrifuged, after which the cells were reconstituted in 100 µL of the same buffer. Subsequently, 5 µL of Annexin-V FITC and 5 µL of propidium iodide (PI) were introduced into the cell suspension, followed by vigorous mixing and a 15 min incubation period at ambient temperature under light-protected conditions. Prior to flow cytometric analysis, the sample volume was adjusted to 500 µL with PBS to facilitate fluorescence detection. Apoptosis data were analyzed by FlowJo Version 10.4 software (Beckman Coulter, Brea, CA, USA).

### 2.13. Mitochondrial Membrane Potential (MMP) Detection

After 12 h of exposure, HCECs were washed with PBS and incubated in MEM medium containing 1 μL of JC-1 at 37 °C for 30 min. The fluorescence of HCECs was measured using fluorescence microscopy. Under high MMP conditions, red fluorescence was produced. Under low MMP, it emitted green fluorescence. The fluorescence emission changed from red to green, indicating MMP reversal.

### 2.14. Detection of SOD, MDA, and CAT

Collected HCECs and tissue samples were lysed by sonication and then the supernatant was gathered by centrifugation. SOD, CAT activities, and MDA levels were measured following the manufacturer’s protocols.

### 2.15. RNA Isolation and Quantitative Real-Time PCR

Total RNA was extracted from HCECs by using Trizol reductant. cDNA was synthesized by preparing a reverse transcription system according to the Promega M-MLVRT instructions. The reaction system for fluorescence quantitative PCR was configured for qRT-PCR using ABI QuantStudio 6 Flex (Invitrogen, Carlsbad, CA, USA), referring to the SYBRR Premix Ex Taq^TM^ instructions.

The relative expression of genes was calculated by the 2^−ΔΔCT^ method using Gapdh as an internal control. The following primers were used in qRT-PCR assays:

*Gapdh*, F: 5′-TGCACCACCAACTGCTTAGC-3′ and R: 5′-GGCATGGACTGTGGTCATGA-3′. *Tnf-α*, F: 5′-GGCGTGGAGCTGAGAGATA-3′ and R: 5′-CAGCCTTGGCCCTTGAAGA-3′. *Il-6*, F: 5′-CAGCCACTCACCTCTTCAGAA-3′ and R: 5′-TGCCTCTTTGCTGCTTTCACA-3′.

### 2.16. Western Blot Analysis

Proteins from HCECs were extracted using a lysis buffer composed of RIPA buffer supplemented with protease and phosphatase inhibitors at a ratio of 100:1:1 (*v*/*v*/*v*). Protein concentrations were determined using a BCA protein quantification kit according to the manufacturer’s protocol. The extracted proteins were separated via SDS-polyacrylamide gel electrophoresis and subsequently transferred onto PVDF membranes. The membranes were blocked with 5% non-fat milk dissolved in TTBS (*m*/*v*) for 2 h at room temperature, followed by incubation with primary antibodies overnight at 4 °C. After thorough washing with TTBS, the membranes were treated with horseradish peroxidase-conjugated secondary antibodies for 1 h at room temperature. Visualization of the target protein bands was achieved using the Immobilon™ Western Chemiluminescent HRP Substrate. Blot images were captured using the ChemiDoc™ XRS+ system (Bio-Rad, Hercules, CA, USA), and the expression levels of the target proteins were quantified with ImageJ software (NIH, USA).

### 2.17. Statistical Analysis

Data were analyzed by using Graphpad Prism 8.0. Values are stated as the mean ± SEM. One-way ANOVA followed by the Tukey test was used to assess differences between more than three groups. The differences were recognized as significant at *p* < 0.05.

## 3. Results

### 3.1. DFC Enhanced Tear Secretion and Reduced Tear Ferning Scale in Mice

**Figure 1:**
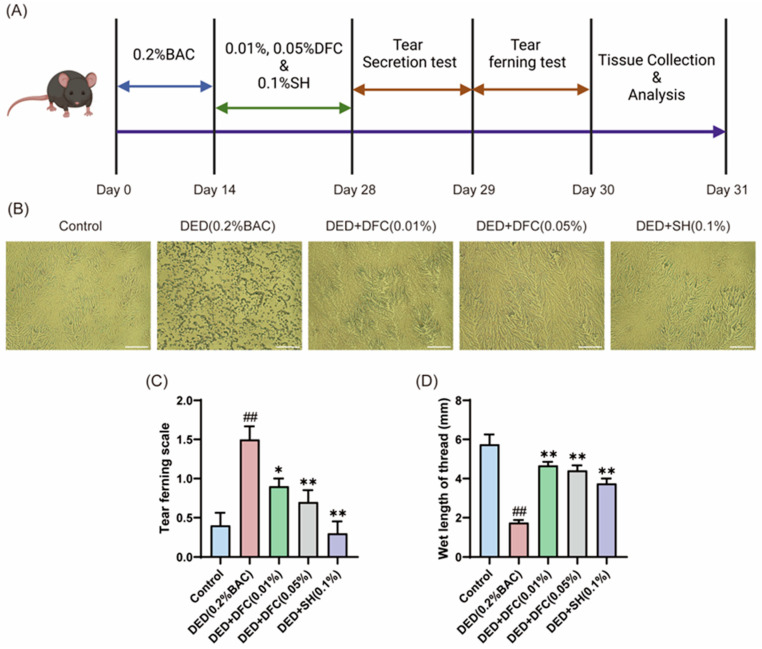
DFC improved the tear secretion function of mice and affected the crystal morphology of lachryllipa. (**A**) Illustration of the experimental procedure. (**B**) Microscopic image of BAC-induced tear fern crystals in DED mice (scale bar = 50 μm). (**C**) Tear fern lens rating results from BAC-induced DED mice (*n* = 10). (**D**) Length of redness of phenolic cotton thread (*n* = 12). Data are shown as mean ± SEM. ^##^ *p* < 0.01 vs. control group; * *p* < 0.05, ** *p* < 0.01 vs. DED group.

On the first day after the completion of the experimental period, the effect of DFC on tear secretion in BAC-induced dry eye mice was tested by phenol cotton thread test. The data showed that the length of cotton thread turning red in the DED group was significantly shorter than that in the control group (*p* < 0.01). The tear secretion of the positive drug SH intervention group was significantly higher than that of the DED group (*p* < 0.01). After treatment with different concentrations of DFC, the Schirmer secretion was significantly increased compared with the DED group (*p* < 0.01) (Figure 1D).

The next day, tears were extracted from the mice and spread on glass slides to observe the fern crystal morphology (Figure 1B). In the control group, the tear fern crystal shape of mice was intact. The tear fern crystals in the DED group were fragmented. After intervention with DFC and SH, the shape of tear fern crystals was significantly restored. The morphology of the tear fern crystals was scored according to the scoring guidelines. The data showed that BAC significantly increased the grading of lachrymofern crystals in the DED group compared with the control group (*p* < 0.01). After intervention with DFC and SH, the grading of tear fern crystals was significantly reduced compared with the DED group (*p* < 0.05, *p* < 0.01).

### 3.2. DFC Prevented the Corneal Epithelial Cell Layers from Thinning

**Figure 2:**
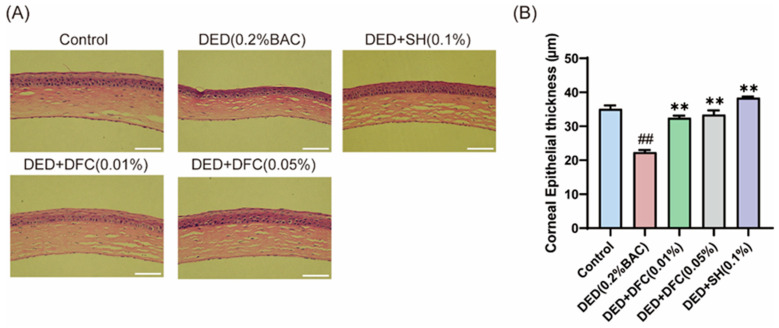
Effect of DFC on corneal thickness in DED mice. (**A**) Representative images of corneal H&E staining of each group of mice (scale bar = 50 μm); (**B**) statistical results of corneal epithelial cell thickness in each group of mice (*n* = 5). Data are shown as mean ± SEM. ^##^ *p* < 0.01 vs. control group; ** *p* < 0.01 vs. DED group.

The ocular surface tissues of the mice were stained with H&E (Figure 2A). The corneal thickness of the mice was measured by Image J, and the results showed that the thickness of the corneal epithelial cell layer in the DED group was significantly reduced compared with the control group (*p* < 0.01). After DFC and SH intervention, the thickness of the corneal epithelial cell layer was significantly increased compared with the DED group (*p* < 0.01) (Figure 2B).

### 3.3. DFC Arrested the Loss of GCs in BAC-Induced DED Mice

**Figure 3:**
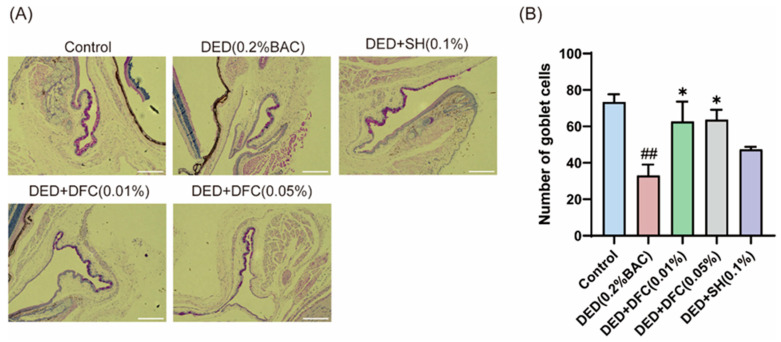
Effect of DFC on GCs in DED mice. (**A**) Representative images of PAS staining in conjunctiva of mice in each group (scale bar = 50 μm); (**B**) statistical results of GCs number in each group of mice (*n* = 3). Data are shown as mean ± SEM. ^##^ *p* < 0.01 vs. control group; * *p* < 0.05 vs. DED group.

The ocular surface tissues of the mice were stained with PAS (Figure 3A). The number of GCs in the conjunctival tissue of the DED group was significantly less than that of the control group (*p* < 0.01) using Image Pro Plus software. After DFC and SH intervention, the number of GCs was significantly increased compared with the DED group (*p* < 0.05) (Figure 3B).

### 3.4. Effects of DFC on Corneal Epithelial Cell Apoptosis in BAC-Induced DED Mice

**Figure 4:**
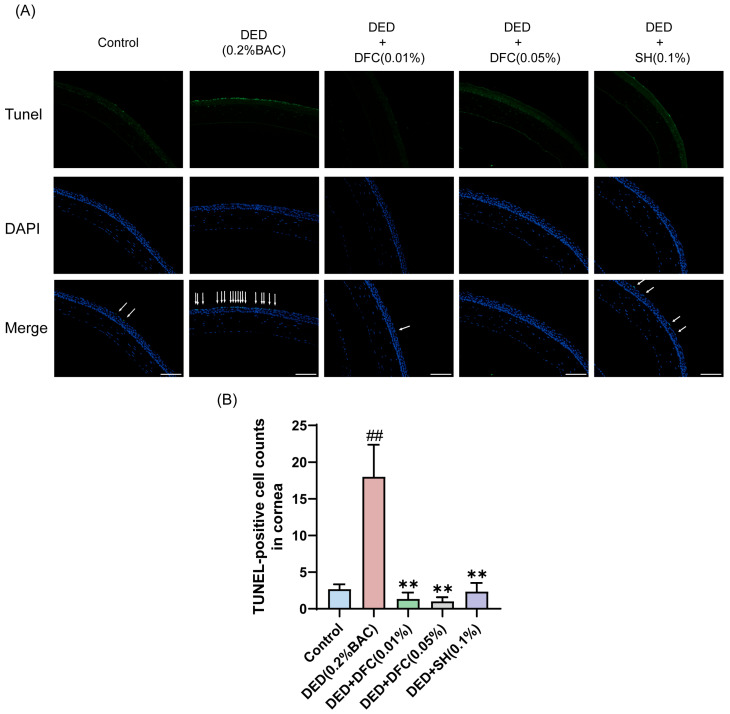
DFC inhibited BAC-induced apoptosis in corneal epithelial cells of DED mice. (**A**) Representative images of TUNEL staining in each group and the white arrows point to the apoptotic corneal epithelial cells in the image (scale bar = 50 μm). (**B**) Results of TUNEL-positive cell count in the cornea (*n* = 3). Data are shown as mean ± SEM. ^##^ *p* < 0.01 vs. control group; ** *p* < 0.01 vs. DED group.

TUNEL staining was performed on mice corneal tissues to detect the effect of DFC on BAC-induced corneal epithelial cell apoptosis in DED mice. Compared with the control group, the number of TUNEL-labeled cells (apoptotic cells) in the corneal epithelium of the DED group was significantly increased (*p* < 0.01). Compared with the DED group, the number of TUNEL-positive cells in mice corneal epithelium was significantly reduced after DFC and SH intervention (*p* < 0.01) (Figure 4A,B).

### 3.5. DFC Enhanced the Cell Viability of HCECs Challenged by NaCl

**Figure 5:**
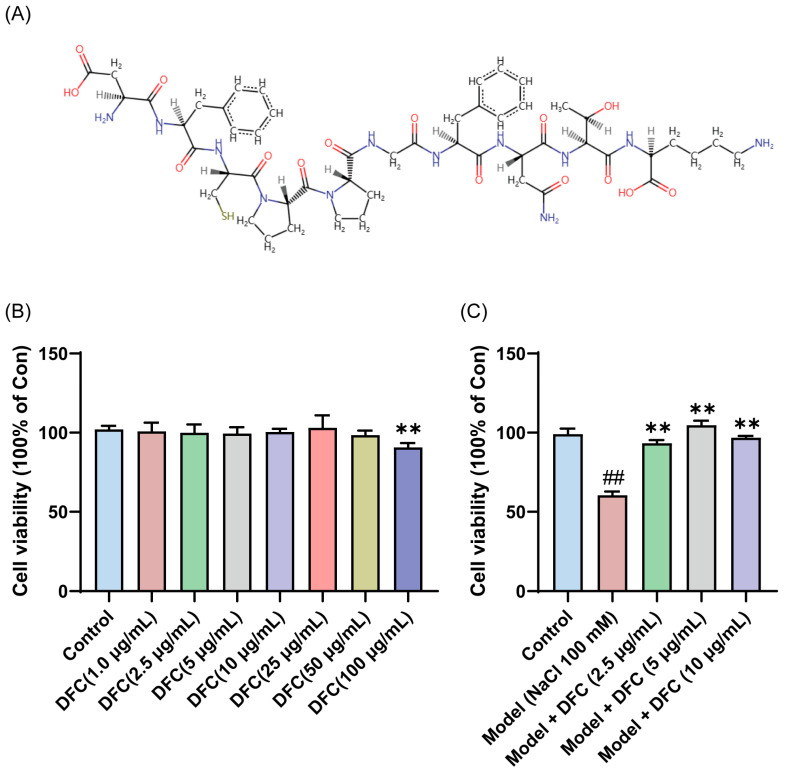
Anti-dry-eye activity of DFC in vitro. (**A**) Molecular structural formula of DFC. (**B**) Cytotoxicity of different concentrations of DFC on HCECs. (**C**) Effects of different concentrations of DFC on NaCl-induced cell viability of HCECs. Data are shown as mean ± SEM (*n* = 4); ^##^ *p* < 0.01 vs. control group; ** *p* < 0.01 vs. model group.

To verify the anti-dry-eye activity of DFC, DFC was synthesized and the sequence information of the synthesized DFC was checked by LC/MS/MS. The results showed that the structural information of the synthesized DFC was correct (Figure S1). The amino acid sequences of the DFC were converted to SMILES format files using the PepSMI (Predicting Protein E (novoprolabs.com, accessed on 15 April 2025)) tool. The SMILES file was uploaded to SwisSTargetPrediction website (http://www.swisstargetprediction.ch, accessed on 15 April 2025) to create the molecular structural formula of DFC (Figure 5A). Compared with the control group, DFC (1, 2.5, 5, 10, 25, and 50 μg/mL, respectively, 12 h) showed no significant cytotoxicity to HCECs, while DFC (100 μg/mL, 12 h) showed significant cytotoxicity to HCECs (*p* < 0.01) (Figure 5B). The cell viability of the model group was only 60.40% of that of the control group, while the cell viability of the DFC (2.5, 5, and 10 μg/mL) treatment groups was significantly higher than that of the model group (*p* < 0.01) (Figure 5C). These results indicate that DFC has a good cytoprotective effect on HCECs induced by NaCl. A significant ameliorative effect on NaCl-induced HCECs viability was observed with DFC at a low concentration of 2.5 μg/mL. Consequently, this concentration was selected for subsequent in vitro experiments.

### 3.6. DFC Inhibited NaCl-Induced Oxidative Stress in HCECs

**Figure 6:**
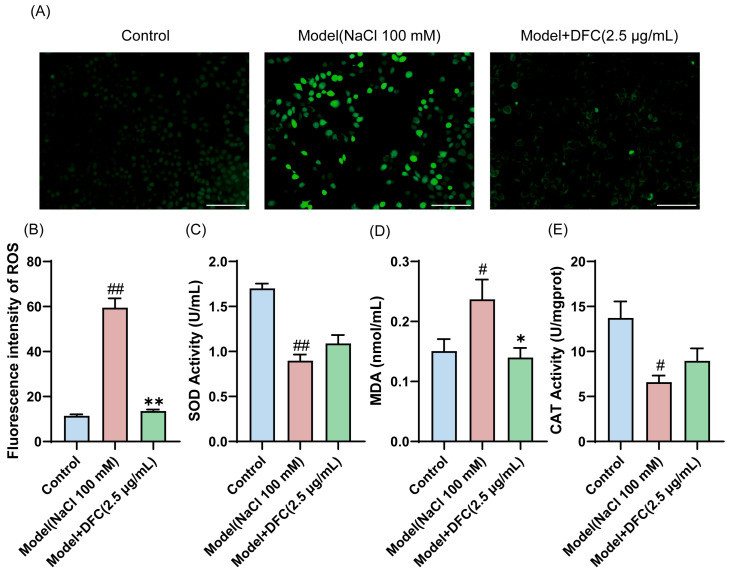
Ability of DFC to resist oxidative stress in response to NaCl stimulation. (**A**) DCFH-DA probe fluorescence microscopy was used to detect ROS production in each group (scale bar = 100 μm). (**B**) Fluorescence intensity quantification in each group (*n* = 3). (**C**–**E**) The levels of oxidative stress factors SOD (**C**), MDA (**D**), and CAT (**E**) were measured in HCECs (*n* = 3). Data are shown as mean ± SEM; ^#^ *p* < 0.05, ^##^ *p* < 0.01 vs. control group; * *p* < 0.05, ** *p* < 0.01 vs. model group.

We examined the ability of DFC to resist oxidative stress. The level of ROS production in HCECs was measured by DCFH-DA staining (Figure 6A). The average intensity of ROS in the model group was significantly increased. The mean intensity of ROS was significantly reduced after DFC intervention (Figure 6B). We also examined the changes in the levels of two of the most important intracellular antioxidant enzymes, SOD and CAT, and MDA, an important indicator of the oxidative system. The results showed that the intracellular MDA content was increased (*p* < 0.05) and the activities of SOD (*p* < 0.01) and CAT (*p* < 0.05) were inhibited by NaCl stimulation. DFC treatment significantly reduced MDA production (*p* < 0.05) and increased SOD and CAT enzyme activities (Figure 6C–E).

### 3.7. DFC Inhibited HCECs Apoptosis Challenged by NaCl

**Figure 7:**
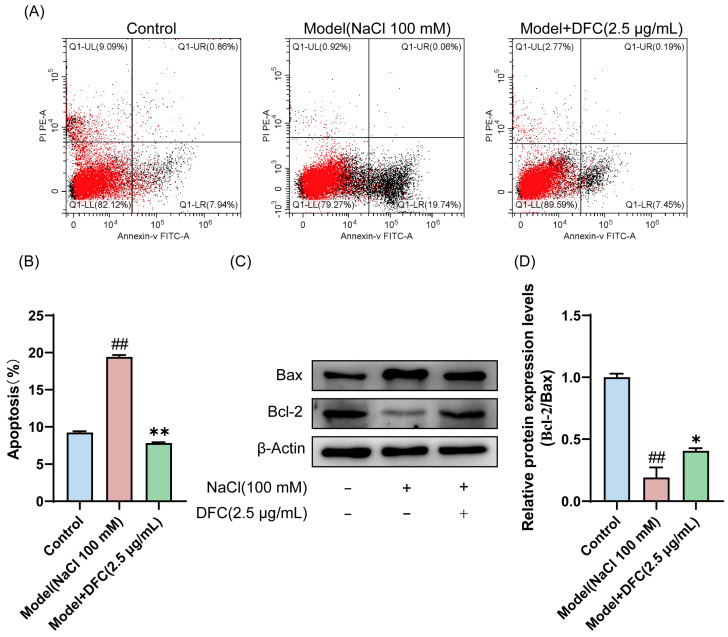
Effect of DFC on NaCl-induced apoptosis of HCECs. (**A**) The apoptosis rate was detected by Annexin-V FITC/PI staining. (**B**) Statistical plot of apoptosis rate was calculated according to UR+LR quadrant (*n* = 3). (**C**,**D**) The protein expression of Bcl-2 and Bax was analyzed by Western blot assay (*n* = 3). Data are shown as mean ± SEM, ^##^ *p* < 0.01 vs. control group; * *p* < 0.05, ** *p* < 0.01 vs. model group.

In the present study, we analyzed the effect of DFC on HCECs’ apoptosis using Annexin-V FITC/PI staining and Western blot. The results of Annexin-V FITC/PI staining showed that, compared with the control group, the apoptosis rate of HCECs in the model group was significantly increased (*p* < 0.01). Compared with the model group, the apoptosis rate of HCECs treated with DFC was significantly decreased (*p* < 0.01) (Figure 7A,B). The expression levels of Bax and Bcl-2 in HCECs were determined by Western blotting (Figure 7C). Compared with the control group, the ratio of Bcl-2/Bax expression level in the model group was significantly decreased (*p* < 0.01). DFC treatment increased the ratio of Bcl-2/Bax expression levels (*p* < 0.05) induced by NaCl in HCECs compared with the model group (Figure 7D).

### 3.8. DFC Restored NaCl-Induced Changes in Membrane Potential of HCECs

**Figure 8:**
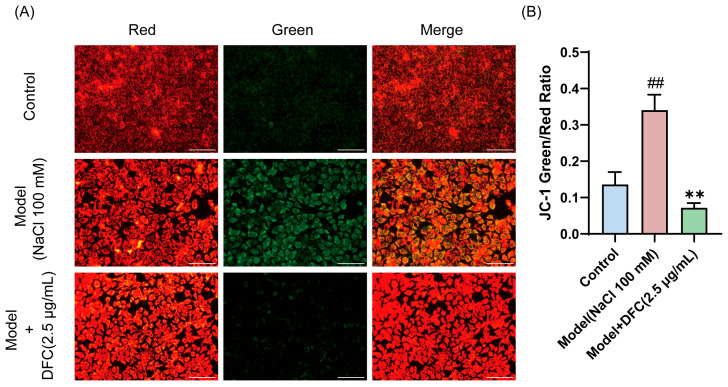
Effect of DFC on mitochondrial membrane potential. (**A**) Fluorescence image of the effect of DFC on NaCl-induced mitochondrial permeability changes in HCECs (scale bar = 100 μm). (**B**) ImageJ fluorescence intensity analysis showing the mean fluorescence intensity of the green/red ratio of JC-1 (*n* = 5). Data are shown as mean ± SEM. ^##^ *p* < 0.01 vs. control group; ** *p* < 0.01 vs. model group.

We observed changes in mitochondrial membrane potential in HCECs, and the decrease in mitochondrial membrane potential is also a hallmark of early apoptosis, as indicated by the transition of JC-1 from red to green fluorescence. JC-1 staining results showed that the fluorescence emission gradually changed from red to green after NaCl stimulation. The fluorescence color transition was significantly reduced after DFC treatment (Figure 8A,B).

### 3.9. DFC Ameliorated NaCl-Induced Inflammation in HCECs

**Figure 9:**
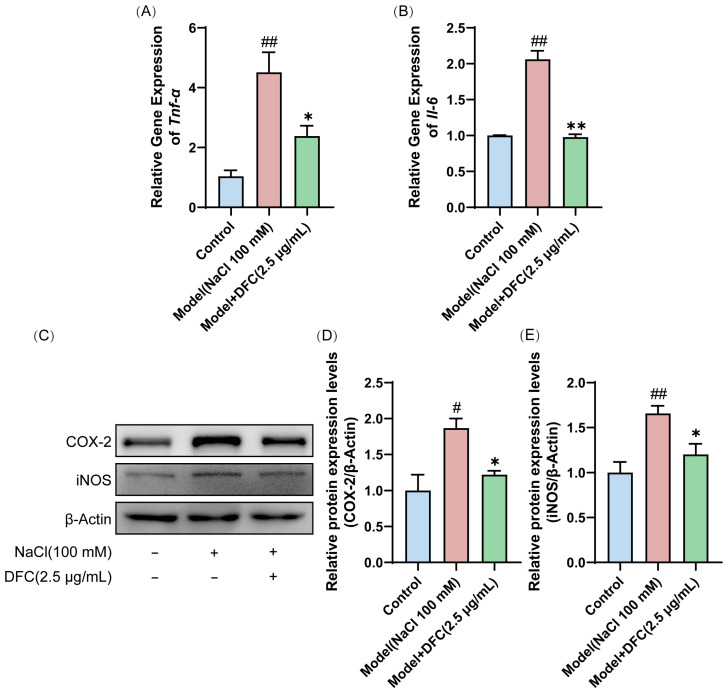
DFC suppressed the inflammatory response in an in vitro dry eye model. The mRNA levels of (**A**) *Tnf-α* and (**B**) *Il-6* were assessed by quantitative real-time PCR analysis (*n* = 3). (**C**) The protein expression levels of COX-2 and iNOS were detected by Western blot. (**D**) Quantification of COX-2 protein expression (*n* = 4). (**E**) Quantification of iNOS protein expression (*n* = 4). Data are shown as mean ± SEM. ^#^ *p* < 0.05, ^##^ *p* < 0.01 vs. control group; * *p* < 0.05, ** *p* < 0.01 vs. model group.

We evaluated the effect of DFC on the mRNA expression of inflammation-related factors *Tnf-α* and *Il-6*. The mRNA expressions of *Tnf-α* and *Il-6* in the model group were significantly higher than those in the control group (*p* < 0.01). After DFC treatment, the mRNA expressions of *Tnf-α* (*p* < 0.05) and *Il-6* (*p* < 0.01) were significantly inhibited (Figure 9A,B). Next, we examined the protein expression levels of COX-2 and iNOS in HCECs by Western blot. Compared with the control group, the expression levels of COX-2 (*p* < 0.05) and iNOS (*p* < 0.01) in the model group were significantly increased. After DFC treatment, the expression levels of COX-2 and iNOS were significantly decreased (*p* < 0.05) compared with the model group (Figure 9C–E).

### 3.10. DFC Modulated the Level of Autophagy in HCECs

**Figure 10:**
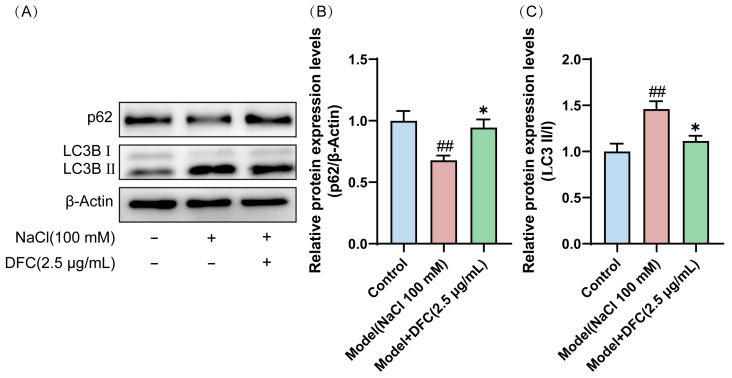
DFC regulated the autophagy level of HCECs induced by NaCl. (**A**) Western blot was used to detect the expression levels of p62 and LC3B proteins. (**B**) Quantification of p62 protein expression (*n* = 5). (**C**) Quantification of LC3B II/I protein expression (*n* = 4). Data are shown as mean ± SEM. ^##^ *p* < 0.01 vs. control group; * *p* < 0.05 vs. model group.

We observed that the conversion of LC3B I protein to the activated form LC3B II was significantly increased in HCECs after NaCl induction (*p* < 0.01). However, after DFC intervention, their expression levels were significantly decreased (*p* < 0.05). Interestingly, the expression level of p62 protein, also known as SQSTM 1, was decreased by NaCl induction (*p* < 0.01) and up-regulated by the addition of DFC intervention (*p* < 0.05) (Figure 10A–C). These results indicated that NaCl stimulation activates autophagy in HCECs and autophagic flux is enhanced by hypertonicity. DFC regulated the autophagy level of HCECs induced by NaCl.

### 3.11. DFC Activated the Nrf2/HO-1 Signaling Pathway in NaCl-Induced HCECs

**Figure 11:**
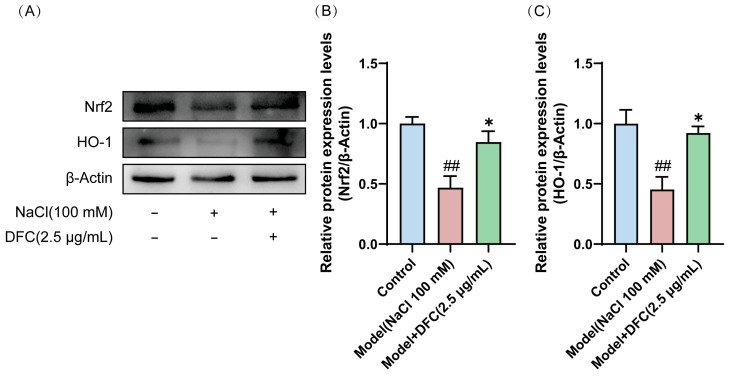
Effects of DFC activated the Nrf2/HO-1 signaling pathway in HCECs induced by NaCl. (**A**) Nrf2 and HO-1 protein expression levels were detected by Western blot. (**B**) Quantification of Nrf2 protein expression (*n* = 4). (**C**) Quantification of HO-1 protein expression (*n* = 4). Data are shown as mean ± SEM. ^##^ *p* < 0.01 vs. control group; * *p* < 0.05 vs. model group.

Western blot showed that the expression levels of Nrf2 and HO-1 proteins in the model group were significantly down-regulated (*p* < 0.01). After DFC treatment, the expression levels of Nrf2 and HO-1 proteins in HCECs were significantly up-regulated compared with the model group (*p* < 0.05) (Figure 11A–C).

## 4. Discussion

As a prevalent ophthalmic disease, the pathogenesis of dry eye is intricate and complex, centering on the reduction in tear secretion, loss of tear film stability, and persistent inflammatory response of the ocular surface [18,19]. With the aging of the population, the popularity of electronic products, and the complexity of environmental factors, the incidence of dry eye has shown a significant increase [20]. Nevertheless, it is not possible for any treatment to provide a complete cure for dry eyes. In recent years, peptides have shown a broad application prospect in the treatment of various diseases due to their unique biological activity and low toxicity [21,22,23]. In a previous study by our group, we found that tilapia skin hydrolysate had ameliorative effects on dry eyes. However, it contains a variety of active peptides, and the specific peptides that can exert anti-dry-eye effects are not known. In the present study, we investigated the potential role and mechanism of DFC peptide to improve dry eye, and DFC showed favorable effects in vitro and in vivo. In vitro, NaCl stimulated HCECs, leading to reduced cell viability, and altered mitochondrial membrane potentials. In vivo, BAC caused mice to develop a number of adverse effects, including decreased tear production, altered tear fern crystal morphology, thinning of the corneal epithelial layer, decreased number of conjunctival goblet cells, and apoptosis of corneal epithelial cells. After the DFC intervention, all of the above symptoms improved significantly.

Multiple studies have demonstrated the role of oxidative stress in the pathogenesis of dry eye disease [24,25,26]. The ocular surface is particularly susceptible to oxidative stress due to its direct exposure to environmental factors. Prolonged contact with ultraviolet (UV) light, air pollutants, and fluctuations in humidity can trigger excessive production of reactive oxygen species (ROS), then induces oxidative stress [24,27]. Oxidative stress occurs when the levels of ROS exceed the antioxidant capacity of corneal epithelial cells due to an imbalance between ROS formed by metabolic reactions and antioxidant defense mechanisms [28]. This contributes to a decline in lacrimal gland function and a decrease in tear secretion [29]. MDA is an endogenous product of lipid peroxidation and is known to react with deoxyribonucleosides to produce a variety of adducts and to damage DNA. In order to protect against the potentially destructive effects of ROS, cells are endowed with a variety of antioxidant enzymes such as superoxide dismutase and catalase, among others [30]. In this study, HCECs stimulated with NaCl showed a significant increase in ROS levels, a decrease in the enzymatic activities of SOD and CAT, and an increase in MDA levels. All oxidative-stress-related indexes improved after DFC intervention, which suggests that DFC can alleviate dry eye by regulating the level of oxidative stress.

Inflammatory response is regarded as the central pathologic mechanism of dry eye [31]. Past studies have confirmed that tear osmolarity is often elevated due to insufficient tear secretion, and hypertonic tears stimulate the release of proinflammatory factors, chemokines, and matrix metalloproteinases (MMPs) from ocular surface epithelial cells, activating schizophrenia-activated protein kinase, nuclear factor κ B, and other signaling pathways, which in turn triggers or exacerbates inflammatory responses [32]. Many studies have shown that MMPs cleave tight junction proteins, occludin, and occludin bandlet-1, leading to corneal epithelial cell detachment [33]. This may be related to the thinning of the corneal epithelial cell layer that we observed in DED mice. The conjunctiva is a fundamental component of the ocular surface. It helps ensure the continued clarification and survival of ocular cells and stroma. Chronic inflammation can lead to conjunctival disease, an important feature of which is loss of conjunctival goblet cells [34]. Inflammation exacerbates the decrease in goblet cells, leading to a lack of mucin in the tear fluid. In addition, tear osmolarity increases with mucin deficiency [35]. Tear hyperosmolality in turn promotes the development of ocular surface inflammation and apoptosis, exacerbating corneal epithelial damage and trapping DED in a vicious cycle [36]. Chronic inflammation can also lead to increased COX-2 and iNOS expression [37,38]. In the present study, COX-2 and iNOS protein expression was up-regulated and mRNA expression of *Tnf-α* and *Il-6* was increased in HCECs by NaCl stimulation. Stimulation of BAC resulted in altered tear fern crystal morphology, decreased corneal epithelial thickness, and decreased conjunctival goblet cells in mice. The above evidence suggests that inflammation is inextricably linked to dry eye. After DFC intervention, all the symptoms related to the above inflammatory responses were significantly alleviated, so it can be assumed that DFC can improve dry eye through its anti-inflammatory effect.

The inflammatory response promotes apoptosis of corneal epithelial cells through inflammatory signaling, and apoptosis is also particularly important in the pathomechanism of dry eye [18]. Tear film stability plays an important role in maintaining the health of the ocular surface and, when there are abnormalities in tear production or changes in osmolality, the tear film’s homeostasis is imbalanced, which leads to apoptosis of the corneal epithelium and a decrease in conjunctival goblet cells [39]. Cells maintain a normal mitochondrial membrane potential on both sides of the inner membrane during normal energy metabolism, whereas mitochondrial dysfunction or a state of early apoptosis can lead to a decrease in mitochondrial membrane potential [40]. Among the major apoptotic signals associated with programed cell death are the up-regulation of Bax and the down-regulation of Bcl-2, which induce cell death, and the modulation of the relative expression of Bcl-2/Bax can regulate apoptosis [41]. In the present study, NaCl stimulation increased the apoptotic rate of HCECs, decreased the mitochondrial membrane potential, and decreased the Bcl-2/Bax ratio. Dry eye mice induced by BAC affected the thickness of the corneal epithelial layer due to corneal epithelial cell detachment and also resulted in a decrease in conjunctival goblet cells. After DFC intervention, all the indexes were ameliorated. Therefore, DFC can improve dry eye by inhibiting apoptosis.

Autophagy is an evolutionarily conserved cellular process that recycles damaged proteins, organelles, and other intracellular materials by delivering them to double-membrane vesicles for lysosomal degradation [42,43]. Autophagy and oxidative stress can regulate each other. Autophagy inhibits the accumulation of ROS by removing damaged mitochondria, whereas oxidative stress occurs when large amounts of ROS are produced, thereby inversely regulating autophagy levels [44,45]. Autophagy is in turn linked to ocular surface inflammation. Autophagy may inhibit the inflammatory response by removing accumulated proteins and maintaining mitochondrial homeostasis, but inadequate autophagy may exacerbate the inflammatory process by decreasing cellular viability and the ability to rapidly eliminate misfolded proteins from the cell [46,47]. LC 3B-II/LC 3B-I is a marker of autophagy. In cells, the cytosolic form of LC 3 (LC3B I) is affixed to phosphatidylethanolamine to form the LC 3-phosphatidylethanolamine affix (LC3B II), which is recruited to the autophagosome membrane. p62 (also known as SQSTM 1) is a selective autophagy receptor and is the most important cargo protein for selective autophagy. This protein serves as a bridge between LC3B and degradation of targeted ubiquitinated substrates. Ubiquitinated proteins that bind p62 enter the autophagosome, where they eventually fuse with lysosomes to form autophagic lysosomes for clearance [48]. When the autophagic stream is activated, the level of p62 decreases. In this study, HCECs were stimulated by NaCl, the level of p62 expression was significantly reduced, and the expression of LC3B II/I was up-regulated. The expression levels of the above proteins were changed after DFC intervention. This suggests that DFC may regulate the level of autophagy in order to participate in the regulation of dry eye development, but the exact underlying mechanism remains to be fully elucidated.

Nrf2/HO-1 is an important pathway that regulates intracellular oxidative stress, inflammation, and apoptosis [49,50]. Nrf2 is an important transcriptional regulator of several protective antioxidant enzymes. When cells are exposed to oxidative stress, Nrf2 translocates to the nucleus and binds to the antioxidant response element (ARE) region, initiating the subsequent transcription of several antioxidant enzymes, including HO-1 [51,52]. Here, we found that DFC treatment significantly increases the protein expression levels of Nrf2 and HO-1. The presented evidence indicates that DFC may enhance the management of dry eye by regulating the Nrf2/HO-1 signaling pathway.

## 5. Conclusions

**Figure 12:**
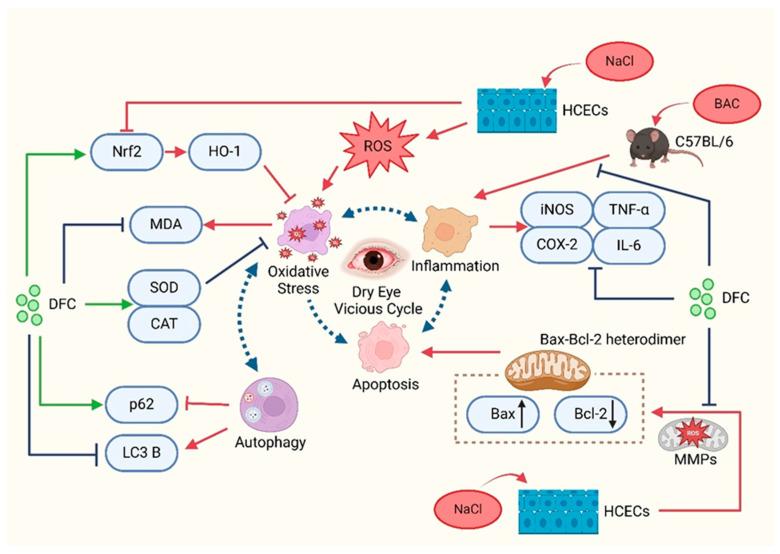
Schematic representation of the mechanisms involved in the improvement of dry eye with DFC.

In summary, the present study demonstrated that DFC can improve dry eye in in vitro and in vivo models. DFC ameliorated the signs of dry eye by inhibiting apoptosis, inflammation, oxidative stress, and modulating the level of autophagy (Figure 12). The findings of this study offer new insights into the prevention and treatment of dry eye, expand the potential for the comprehensive utilization of aquatic waste, and confirm the novel active peptide DFC as a promising avenue for dry eye treatment.

## Data Availability

The data presented in this study are available on request from the corresponding authors.

## Supplementary Materials

The following supporting information can be downloaded at: https://www.mdpi.com/article/10.3390/cimb47060441/s1.

## Abbreviations

The following abbreviations are used in this manuscript:

BAC	Benzalkonium chloride
Bax	Bcl-2-associated X protein
BCA	Bicinchoninic acid
Bcl-2	B-cell lymphoma 2
CAT	Catalase
COX-2	Anti-cyclooxygenase-2
DAPI	4,6-diamidino-2-phenylindole
DED	Dry eye disease
DCFH-DA	2′,7′-dichlorofluorescein diacetate
GCs	Goblet cells
HCECs	Human corneal epithelial cells
H&E	Hematoxylin and eosin
HO-1	Heme oxygenase-1
IL-6	Interleukin-6
iNOS	Inducible nitric oxide synthase
LC3B	Microtubule-associated protein 1 light chain 3 beta
MDA	Malondialdehyde
MEM	Modified Eagle’s medium
MMP	Mitochondrial membrane potential
MMPs	Matrix metalloproteinases
Nrf2	Nuclear factor E2-related factor
PAS	Periodic acid-schiff
PBS	Fetal bovine serum
ROS	Reactive oxygen species
SH	Sodium Hyaluronate
SOD	Superoxide dismutase
SQSTM1	Sequestosome-1
TDT	Terminal deoxynucleotidyl transferase
TNF-α	Tumor necrosis factor-alpha
TSP	Tilapia skin peptide
TUNEL	Transferase-mediated dUTP Nick-End Labeling labeling

## References

[1] Craig J.P., Nichols K.K., Akpek E.K., Caffery B., Dua H.S., Joo C.K., Liu Z., Nelson J.D., Nichols J.J., Tsubota K. (2017). TFOS DEWS II Definition and Classification Report. Ocul. Surf..

[2] Walter K. (2022). What Is Dry Eye Disease?. JAMA.

[3] Li S., Lu Z., Huang Y., Wang Y., Jin Q., Shentu X., Ye J., Ji J., Yao K., Han H. (2022). Anti-Oxidative and Anti-Inflammatory Micelles: Break the Dry Eye Vicious Cycle. Adv. Sci..

[4] Labetoulle M., Benitez-del-Castillo J.M., Barabino S., Herrero Vanrell R., Daull P., Garrigue J.-S., Rolando M. (2022). Artificial Tears: Biological Role of Their Ingredients in the Management of Dry Eye Disease. Int. J. Mol. Sci..

[5] Wang Y., Chen X.-S. (2023). Recent advance in natural plant products for treatment of dry eye disease. TMR Integr. Med..

[6] Abengózar-Vela A., Calonge M., Stern M.E., González-García M.J., Enríquez-De-Salamanca A. (2015). Quercetin and resveratrol decrease the inflammatory and oxidative responses in human ocular surface epithelial cells. Investig. Ophthalmol. Vis. Sci..

[7] Shetty R., Subramani M., Murugeswari P., Anandula V.R., Matalia H., Jayadev C., Ghosh A., Das D. (2020). Resveratrol Rescues Human Corneal Epithelial Cells Cultured in Hyperosmolar Conditions: Potential for Dry Eye Disease Treatment. Cornea.

[8] McKay T.B., Karamichos D. (2017). Quercetin and the ocular surface: What we know and where we are going. Exp. Biol. Med..

[9] Muz O.E., Orhan C., Erten F., Tuzcu M., Ozercan I.H., Singh P., Morde A., Padigaru M., Rai D., Sahin K. (2020). A novel integrated active herbal formulation ameliorates dry eye syndrome by inhibiting inflammation and oxidative stress and enhancing glycosylated phosphoproteins in rats. Pharmaceuticals.

[10] de Castro R.J.S., Sato H.H. (2015). Biologically active peptides: Processes for their generation, purification and identification and applications as natural additives in the food and pharmaceutical industries. Food Res. Int..

[11] Pescina S., Ostacolo C., Gomez-Monterrey I.M., Sala M., Bertamino A., Sonvico F., Padula C., Santi P., Bianchera A., Nicoli S. (2018). Cell penetrating peptides in ocular drug delivery: State of the art. J. Control Release.

[12] Chen S., Barnstable C.J., Zhang X., Li X., Zhao S., Tombran-Tink J. (2024). A PEDF peptide mimetic effectively relieves dry eye in a diabetic murine model by restoring corneal nerve, barrier, and lacrimal gland function. Ocul. Surf..

[13] Ho T.-C., Fan N.-W., Yeh S.-I., Chen S.-L., Tsao Y.-P. (2022). The Therapeutic Effects of a PEDF-Derived Short Peptide on Murine Experimental Dry Eye Involves Suppression of MMP-9 and Inflammation. Transl. Vis. Sci. Technol..

[14] Zeng J., Lin C., Zhang S., Yin H., Deng K., Yang Z., Zhang Y., Liu Y., Hu C., Zhao Y.T. (2023). Isolation and Identification of a Novel Anti-Dry Eye Peptide from Tilapia Skin Peptides Based on In Silico, In Vitro, and In Vivo Approaches. Int. J. Mol. Sci..

[15] Zeng J., Hu C., Lin C., Zhang S., Deng K., Du J., Yang Z., Liu S., Wu W., Zhao Y.-T. (2023). Tilapia Skin Peptides Inhibit Apoptosis, Inflammation, and Oxidative Stress to Improve Dry Eye Disease In Vitro and In Vivo. J. Food Biochem..

[16] Xiao X., He H., Lin Z., Luo P., He H., Zhou T., Zhou Y., Liu Z. (2012). Therapeutic effects of epidermal growth factor on benzalkonium chloride-induced dry eye in a mouse model. Investig. Ophthalmol. Vis. Sci..

[17] Tang Y.-J., Chang H.-H., Tsai C.-Y., Chen L.-Y., Lin D.P.-C. (2020). Establishment of a Tear Ferning Test Protocol in the Mouse Model. Transl. Vis. Sci. Technol..

[18] Yu L., Yu C., Dong H., Mu Y., Zhang R., Zhang Q., Liang W., Li W., Wang X., Zhang L. (2021). Recent developments about the pathogenesis of dry eye disease: Based on immune inflammatory mechanisms. Front. Pharmacol..

[19] Barabino S., Dana M.R. (2004). Animal models of dry eye: A critical assessment of opportunities and limitations. Investig. Ophthalmol. Vis. Sci..

[20] Britten-Jones A.C., Wang M.T.M., Samuels I., Jennings C., Stapleton F., Craig J.P. (2024). Epidemiology and Risk Factors of Dry Eye Disease: Considerations for Clinical Management. Medicina.

[21] Liu H., Zhang L., Yu J., Shao S. (2024). Advances in the application and mechanism of bioactive peptides in the treatment of inflammation. Front. Immunol..

[22] Akbarian M., Khani A., Eghbalpour S., Uversky V.N. (2022). Bioactive Peptides: Synthesis, Sources, Applications, and Proposed Mechanisms of Action. Int. J. Mol. Sci..

[23] Bouglé D., Bouhallab S. (2017). Dietary bioactive peptides: Human studies. Crit. Rev. Food Sci. Nutr..

[24] Böhm E.W., Buonfiglio F., Voigt A.M., Bachmann P., Safi T., Pfeiffer N., Gericke A. (2023). Oxidative stress in the eye and its role in the pathophysiology of ocular diseases. Redox Biol..

[25] Dogru M., Kojima T., Simsek C., Tsubota K. (2018). Potential Role of Oxidative Stress in Ocular Surface Inflammation and Dry Eye Disease. Investig. Ophthalmol. Vis. Sci..

[26] Seen S., Tong L. (2018). Dry eye disease and oxidative stress. Acta Ophthalmol..

[27] Rauchman S.H., Locke B., Albert J., De Leon J., Peltier M.R., Reiss A.B. (2023). Toxic External Exposure Leading to Ocular Surface Injury. Vision.

[28] Finkel T. (1998). Oxygen radicals and signaling. Curr. Opin. Cell Biol..

[29] Uchino Y., Kawakita T., Miyazawa M., Ishii T., Onouchi H., Yasuda K., Ogawa Y., Shimmura S., Ishii N., Tsubota K. (2012). Oxidative stress induced inflammation initiates functional decline of tear production. PLoS ONE.

[30] Deng R., Hua X., Li J., Chi W., Zhang Z., Lu F., Zhang L., Pflugfelder S.C., Li D.-Q. (2015). Oxidative stress markers induced by hyperosmolarity in primary human corneal epithelial cells. PLoS ONE.

[31] Chu L., Wang C., Zhou H. (2024). Inflammation mechanism and anti-inflammatory therapy of dry eye. Front. Med..

[32] Harrell C.R., Feulner L., Djonov V., Pavlovic D., Volarevic V. (2023). The Molecular Mechanisms Responsible for Tear Hyperosmolarity-Induced Pathological Changes in the Eyes of Dry Eye Disease Patients. Cells.

[33] Zhang Y., Yang M., Zhao S.-X., Nie L., Shen L.-J., Han W. (2022). Hyperosmolarity disrupts tight junction via TNF-α/MMP pathway in primary human corneal epithelial cells. Int. J. Ophthalmol..

[34] Dartt D.A., Masli S. (2014). Conjunctival epithelial and goblet cell function in chronic inflammation and ocular allergic inflammation. Curr. Opin. Allergy Clin. Immunol..

[35] Alam J., de Paiva C.S., Pflugfelder S.C. (2020). Immune-Goblet cell interaction in the conjunctiva. Ocul. Surf..

[36] Perez V.L., Stern M.E., Pflugfelder S.C. (2020). Inflammatory basis for dry eye disease flares. Exp. Eye Res..

[37] Minghetti L. (2004). Cyclooxygenase-2 (COX-2) in inflammatory and degenerative brain diseases. J. Neuropathol. Exp. Neurol..

[38] Needleman P., Manning P.T. (1999). Interactions between the inducible cyclooxygenase (COX-2) and nitric oxide synthase (iNOS) pathways: Implications for therapeutic intervention in osteoarthritis. Osteoarthr. Cartil..

[39] Zhang X., Jeyalatha M V., Qu Y., He X., Ou S., Bu J., Jia C., Wang J., Wu H., Liu Z. (2017). Dry Eye Management: Targeting the Ocular Surface Microenvironment. Int. J. Mol. Sci..

[40] Zorova L.D., Popkov V.A., Plotnikov E.Y., Silachev D.N., Pevzner I.B., Jankauskas S.S., Babenko V.A., Zorov S.D., Balakireva A.V., Juhaszova M. (2018). Mitochondrial membrane potential. Anal. Biochem..

[41] Basu A., Haldar S. (1998). The relationship between BcI2, Bax and p53: Consequences for cell cycle progression and cell death. Mol. Hum. Reprod..

[42] Parzych K.R., Klionsky D.J. (2014). An overview of autophagy: Morphology, mechanism, and regulation. Antioxid. Redox Signal..

[43] Ryter S.W., Cloonan S.M., Choi A.M.K. (2013). Autophagy: A critical regulator of cellular metabolism and homeostasis. Mol. Cells.

[44] Filomeni G., De Zio D., Cecconi F. (2015). Oxidative stress and autophagy: The clash between damage and metabolic needs. Cell Death Differ..

[45] Ornatowski W., Lu Q., Yegambaram M., Garcia A.E., Zemskov E.A., Maltepe E., Fineman J.R., Wang T., Black S.M. (2020). Complex interplay between autophagy and oxidative stress in the development of pulmonary disease. Redox Biol..

[46] Jeyabalan N., Pillai A.M., Khamar P., Shetty R., Mohan R.R., Ghosh A. (2023). Autophagy in dry eye disease: Therapeutic implications of autophagy modulators on the ocular surface. Indian J. Ophthalmol..

[47] Liu Z., Chen D., Chen X., Bian F., Gao N., Li J., Pflugfelder S.C., Li D.-Q. (2020). Autophagy Activation Protects Ocular Surface from Inflammation in a Dry Eye Model In Vitro. Int. J. Mol. Sci..

[48] Lippai M., Lőw P. (2014). The role of the selective adaptor p62 and ubiquitin-like proteins in autophagy. BioMed Res. Int..

[49] Loboda A., Damulewicz M., Pyza E., Jozkowicz A., Dulak J. (2016). Role of Nrf2/HO-1 system in development, oxidative stress response and diseases: An evolutionarily conserved mechanism. Cell. Mol. Life Sci..

[50] Zhang Q., Liu J., Duan H., Li R., Peng W., Wu C. (2021). Activation of Nrf2/HO-1 signaling: An important molecular mechanism of herbal medicine in the treatment of atherosclerosis via the protection of vascular endothelial cells from oxidative stress. J. Adv. Res..

[51] Tu W., Wang H., Li S., Liu Q., Sha H. (2019). The Anti-Inflammatory and Anti-Oxidant Mechanisms of the Keap1/Nrf2/ARE Signaling Pathway in Chronic Diseases. Aging Dis..

[52] Jeong J.Y., Cha H.J., Choi E.O., Kim C.H., Kim G.Y., Yoo Y.H., Hwang H.J., Park H.T., Yoon H.M., Choi Y.H. (2019). Activation of the Nrf2/HO-1 signaling pathway contributes to the protective effects of baicalein against oxidative stress-induced DNA damage and apoptosis in HEI193 Schwann cells. Int. J. Med. Sci..

