# Supplementary material for: Peptide DFCPPGFNTK Mitigates Dry Eye Pathophysiology by Suppressing Oxidative Stress, Apoptosis, Inflammation, and Autophagy: Evidence from In Vitro and In Vivo Models
Source: Curr Issues Mol Biol. 2025 Jun 10;47(6):441. doi: 10.3390/cimb47060441 (PMC12191653; doi:10.3390/cimb47060441)
Supplement: Supplementary file 1 [file cimb-47-00441-s001.zip › cimb-3619662-supplementary.pdf]

(A)

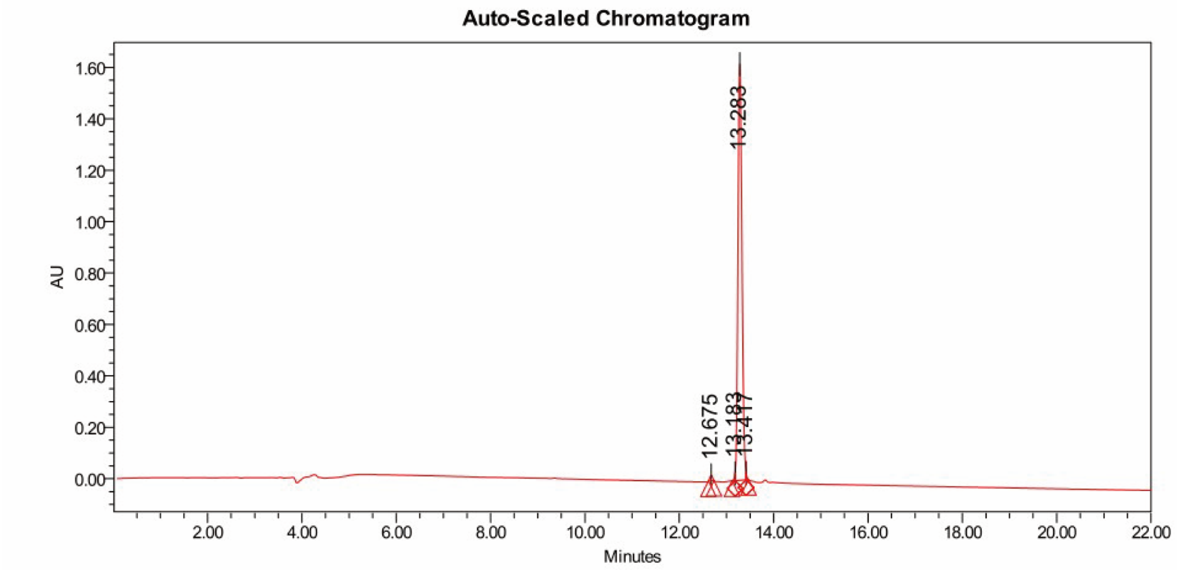

Peak Results

	RT	Area	Height	% Area
1	12.675	81985	21149	0.86
2	13.183	32641	28750	0.34
3	13.283	9414342	1612414	98.48
4	13.417	30895	26644	0.32

(B)

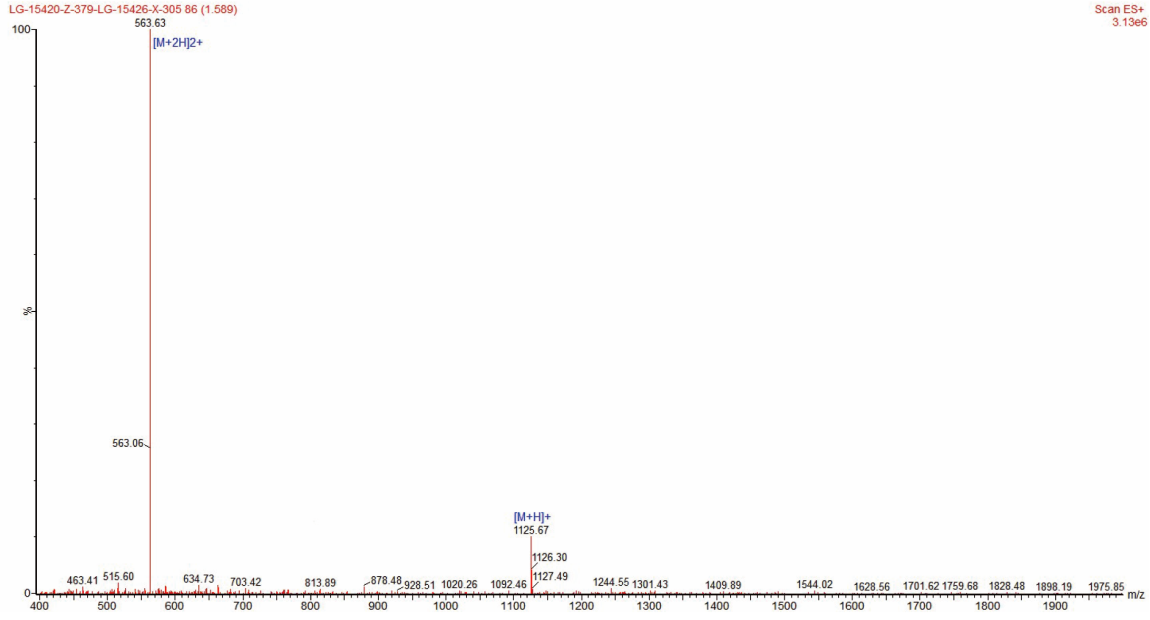

Figure S1. Results of HPLC/MS assay of DFC. (**A**) HPLC results of DFC; (**B**) MS results of DFC.
